# Comprehensive profiling identifies a novel signature with robust predictive value and reveals the potential drug resistance mechanism in glioma

**DOI:** 10.1186/s12964-019-0492-6

**Published:** 2020-01-06

**Authors:** Fan Zeng, Kuanyu Wang, Xiu Liu, Zheng Zhao

**Affiliations:** 1 Department of Molecular Neuropathology, Beijing Neurosurgical Institute, Capital Medical University, No.119 South 4th Ring Road West, Fengtai District, Beijing, 100070 China; 2 Department of Neurosurgery, Beijing Neurosurgical Institute, Capital Medical University, Beijing, 100070 China

**Keywords:** Glioma, Tumor stemness, Drug resistance, Gene signature, Prognosis

## Abstract

**Background:**

Gliomas are the most common and malignant brain tumors. The standard therapy is surgery combined with radiotherapy, chemotherapy, and/or other comprehensive methods. However, the emergence of chemoresistance is the main obstacle in treatment and its mechanism is still unclear.

**Methods:**

We firstly developed a multi-gene signature by integrated analysis of cancer stem cell and drug resistance related genes. The Chinese Glioma Genome Atlas (CGGA, 325 samples) and The Cancer Genome Atlas (TCGA, 699 samples) datasets were then employed to verify the efficacy of the risk signature and investigate its significance in glioma prognosis. GraphPad Prism, SPSS and R language were used for statistical analysis and graphical work.

**Results:**

This signature could distinguish the prognosis of patients, and patients with high risk score exhibited short survival time. The Cox regression and Nomogram model indicated the independent prognostic performance and high prognostic accuracy of the signature for survival. Combined with a well-known chemotherapy impact factor-MGMT promoter methylation status, this risk signature could further subdivide patients with distinct survival. Functional analysis of associated genes revealed signature-related biological process of cell proliferation, immune response and cell stemness. These mechanisms were confirmed in patient samples.

**Conclusions:**

The signature was an independent and powerful prognostic biomarker in glioma, which would improve risk stratification and provide a more accurate assessment of personalized treatment.

Additional file 8 Video abstract

## Background

Glioma is the most common primary malignant tumors of the central nervous system, accounting for 30% of all brain tumors and 80% of all malignant brain tumors [1]. It is one of the representative malignant tumors with the characteristics of strong genetic heterogeneity, high mortality and chemotherapy resistance. According to the 2016 WHO classification of central nervous system (CNS) tumors, the diffuse gliomas are mainly divided into five subtypes based on the mutation status of isocitrate dehydrogenase (IDH) and Chromosome 1p/19q status, namely low-grade gliomas (LGG) with IDH-mutant and 1p/19q-intact subtype, IDH-mutant and 1p/19q-codeleted subtype, LGG with IDH-wildtype subtype, glioblastoma (GBM) with IDH-mutant subtype, and GBM with IDH-wildtype subtype [2]. This classification breaks the principle of diagnosis based entirely on histological features and incorporates genetic parameters into the classification of CNS tumors. Therefore, the further exploration of novel and reliable biomarkers for the prediction of glioma may help to elucidate the molecular mechanism of glioma development and progression.

Temozolomide (TMZ), the most commonly used drug for standard clinical chemotherapy, has improved both the overall survival (OS) and the progression-free survival of patients in glioma. However, the patients usually suffer drug resistance and the underlying mechanism is still elusive. Recently, researchers have found the relationship between cancer stem cells (CSCs) and drug resistance. The CSCs represent a rare population of cancer cells with the capacity to self-renew and initiate tumors. Several biomarkers or relevant molecular markers have been reported differentially expressed in CSCs, suggesting the stemness and unfavorable prognosis [3, 4]. Even therapies that cause complete regression of tumors might spare enough CSCs to allow regrowth of the tumors [5, 6]. Currently, the research on CSC-associated drug resistance is limited, and no method has been established to predict drug resistance through the expression of CSC-related genes, and ultimately predict the prognosis of patients. Therefore, we focused on TMZ therapy, CSCs and diverse related oncogenic drivers within patients by high throughput sequencing method and established gene signature, a statistical model including several biomarkers, to predict the prognosis and chemoresistance of glioma patients [7–9].

In this study, we developed and validated a multigene signature including CSCs and TMZ drug resistant (TMZ-DR)-associated genes to predict patients’ prognosis in glioma. Multivariate analysis revealed the correlation between gene signature and malignant progression of cancer and further clarified the potential biological mechanisms. Combined with the methylation state of O^6^-methylguanine-DNA methyltransferase (MGMT) promoter, this gene signature could more comprehensively predict the prognosis of patients, exhibiting impressive clinical application value. In addition, we validated the signature in GBM patients that only treated with TMZ in the Chinese Glioma Genome Atlas Network (CGGA) dataset and elucidated the mechanism of TMZ drug resistance to some extent. Our result suggested that the signature integrating comprehensive transcript information would improve risk stratification and provide a more accurate assessment of personalized clinical management in patients with glioma. These results might provide new view for glioma malignancy and individual treatment.

## Materials and methods

### Patients and databases

A total of 325 glioma samples from the Chinese Glioma Genome Atlas Network (CGGA, http://www.cgga.org.cn) were included in this study [10]. All these samples and clinicopathological information were collected with informed consent. The study was approved by the Tiantan Hospital Institutional Review Board and kept consistent with the principles of the Helsinki Declaration. An independent cohort of 699 patients with clinical and molecular profiling was obtained from TCGA (http://cancergenome.nih.gov) and used as external validation. The GSE23806 dataset from GEO website (https://www.ncbi.nlm.nih.gov/geo/) was downloaded, including 27 glioma stem-like cell lines and 36 conventional glioma cell lines, and used to discover the CSC-associated genes. Besides, a drug screening profile (COSMIC dataset) was obtained from the public website (https://cancer.sanger.ac.uk/cell_lines/) to do the drug sensibility analysis.

### Development of signature and analytical approach

The student’s t-test was first performed to identify differentially expressed genes in cancer stem cells (GSE23806 dataset) and TMZ-resistant cells (COSMIC dataset) respectively. The common differential genes were then dimensionally reduced by the least absolute shrinkage and selection operator (LASSO) method. These obtained genes finally formed a risk signature that was determined by a linear combination of their expression levels weighted with regression coefficients from univariate Cox regression analyses. The hazard ratio (HR) of each gene figured out in CGGA dataset was used to develop the signature:

$$ Signature\kern0.17em risk\kern0.17em score={\sum}_{i=1}^n{\beta}_i{x}_i $$

where βi indicates the HR for each gene, and *x*_*i*_ indicates the z score transformed relative expression value of each gene.

The Kaplan-Meier survival curves were used to estimate survival distributions. Cox regression was performed to assess the prognostic value of the risk score. The DAVID software (http://david.ncifcrf.gov/) was applied to elucidate the Gene Ontology (GO) biological functions and KEGG pathway. The Gene Set Enrichment Analysis (GSEA, http://www.broadinstitute.org/gsea/index.jsp) was performed to identify gene sets of statistical difference between two groups (high risk score vs. low risk score). Figures were generated by several packages of R software (version 3.2.5), such as ‘pheatmap’, ‘pROC’, and ‘circlize’ [11, 12].

### Immunohistochemistry

To verify the significance and potential mechanism of the risk signature, we analyzed immunohistochemical (IHC) protein staining data of CD133, P4HB, IBA1 and CD163 in the glioma samples from CGGA dataset. The IHC expression levels were compared in the low-, medium- and high-risk score groups with a nonparametric test. Briefly, five-micrometer-thick sections were deparaffinized, boiled with EDTA antigen retrieval buffer, and then incubated with the primary antibodies overnight at 4 °C (anti-CD133 antibody, 1:1000 dilution, Proteintech Group; anti-P4HB, 1:1000, Abcam; anti-IBA1, 1:2000, Abcam; anti-CD163, 1:200, Abcam). Then, the sections were incubated with appropriate secondary antibodies (1:100, ZSGB-Bio, Beijing, China) at room temperature for 1 h. Finally, the stained slides were individually reviewed and evaluated by two investigators. The expression levels of each protein in tumor tissues were defined as the portion of positively stained cells against total counted cells. The difference was assessed by Student-t test.

### Construction of an individualized prediction model

A nomogram was established as an individualized prediction model to predict patient’s overall survival using the ‘rms’ package in R language software [13]. The final model was constructed using a backward step-down selection process based on the Akaike information criterion. Concordance index (C-index) and calibration curves were performed to assess predictive accuracy and discriminative ability of the nomogram. The prognostic nomogram model was estimated in the training cohort and then tested in the validation cohort.

### Statistical analysis

All statistical analyses were conducted using R language (version 3.4.1), GraphPad Prism (version 7.0) and SPSS (version 16.0). The Student’s t-test was used to compare the differences in variables between groups. *P* < 0.05 was regarded as statistically significant.

## Results

### Gene selection and signature building

**Fig. 1:**
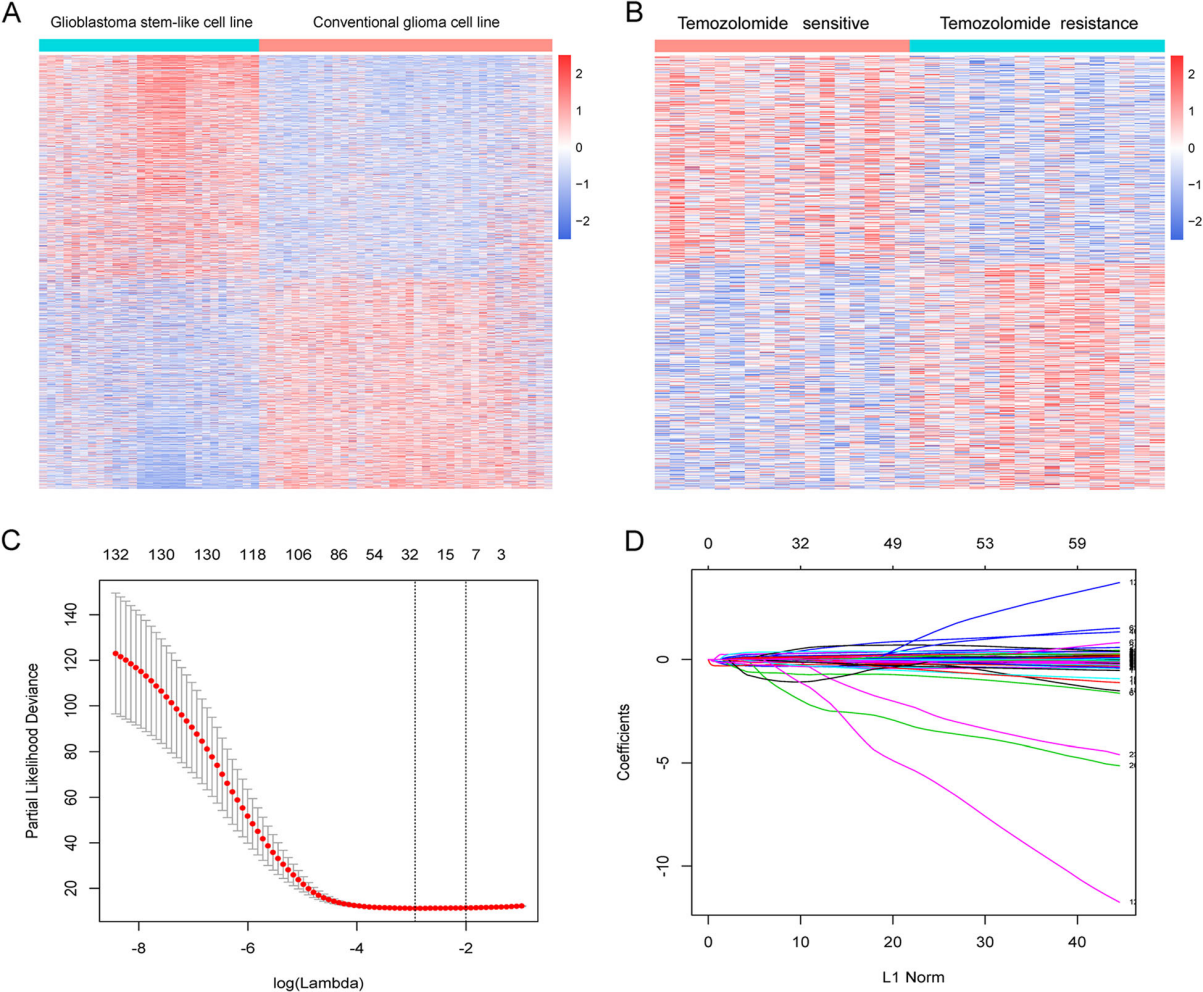
Identification and establishment of gene signature. **a** The differentially expressed genes between CSCs and conventional glioma cell lines. **b** The differentially expressed genes between TMZ sensitive cell lines and TMZ resistant cell lines. **c** Ten-time cross-validation for tuning parameter selection in the LASSO model. **d** LASSO coefficient profiles

CSCs are subpopulations of cancer cells with unlimited self-renewal potential that drive tumorigenesis and exhibit resistance to chemotherapeutics [14, 15]. Elimination of CSCs is the key step to overcome drug resistance. Therefore, our study starts with the analysis of CSCs. The student’s t-test was carried out in GSE23806 dataset to identify the different expression genes between glioma stem-like cell lines (*n* = 27) and conventional glioma cell lines (*n* = 36) (*P* < 0.05). A total of 6752 genes were highly expressed in glioma stem-like cell lines and were selected for further analysis (Fig. 1a). The same analysis was applied in COSMIC dataset to reveal the TMZ-DR genes according to the half maximal inhibitory concentration (IC_50_). A total of 447 genes were identified highly expressed in the high IC_50_ group (Fig. 1b). To select bona fide drug-resistant associated genes, 138 genes were identified using intersections of above two gene sets. Lastly, the LASSO Cox regression model was conducted to select the most useful predictive features and identified only seven genes (*ATL1, GRIA3, HPX, IL17D, KLHDC1, NCAM2, TRIM67*) with non-zero regression coefficients (Fig. 1c and d) in CGGA datasets.

We then established a signature for glioma patients based on the gene expression levels as follows: signature risk score = (− 0.143 × ATL1 expression) + (− 0.094 × GRIA3 expression) + (0.171 × HPX expression) + (− 0.305 × IL17D expression) + (− 0.132 × KLHDC1 expression) + (− 0.096 × NCAM2 expression) + (− 0.124 × TRIM67 expression). The coefficients of each gene indicate the HR. The coefficient of HPX is greater than 1, implying its tumor driving characteristic, while the coefficients of the other four genes are less than 1, revealing their protecting effect.

### Molecular characteristics of the risk signature

**Fig. 2:**
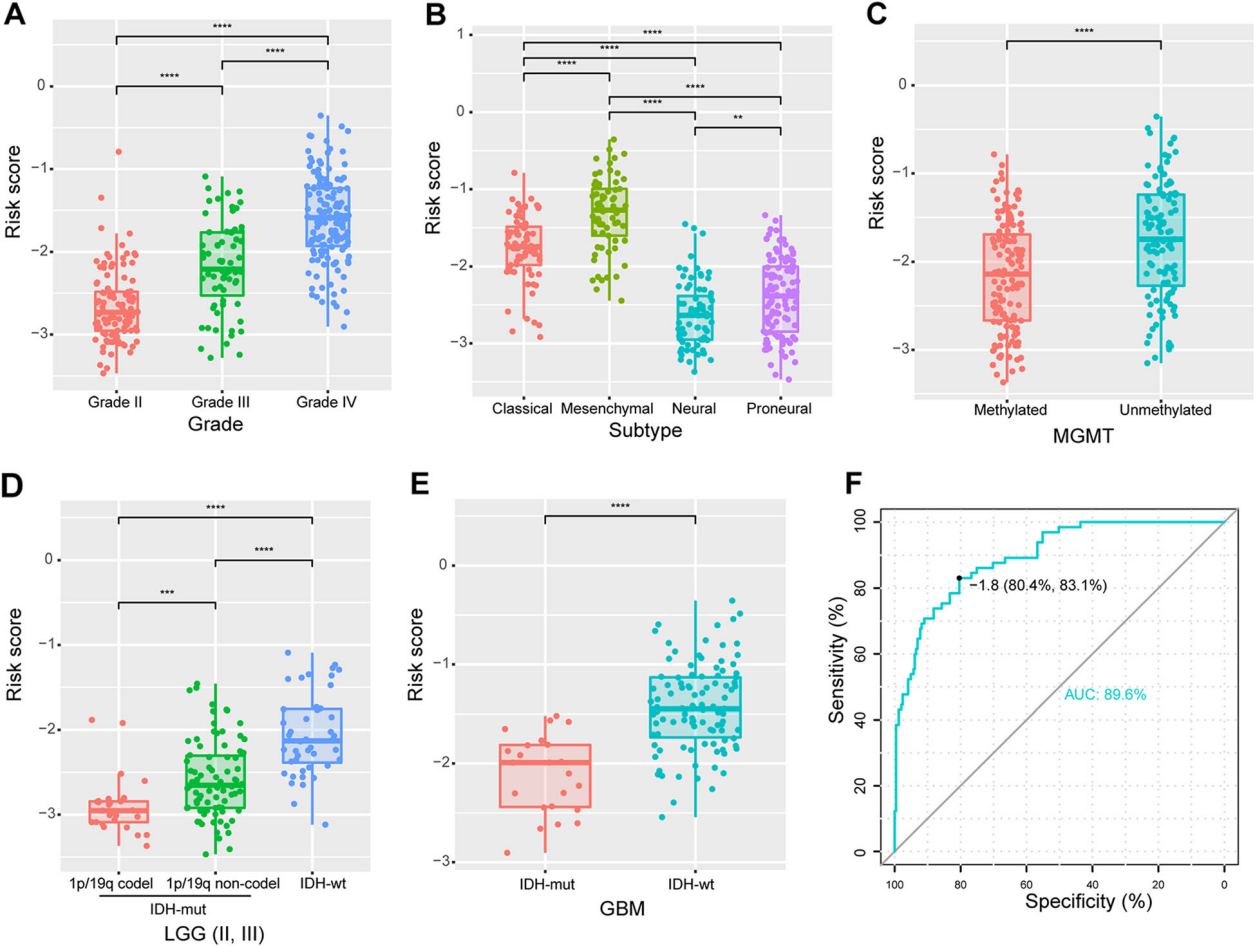
Molecular characteristics and risk score distribution in patients from CGGA dataset. **a** Risk score distribution among different pathologic grades. **b** Risk score distribution among different subtypes of patients. **c** Risk score distribution between MGMT promoter methylated and unmethylated patients. **d**-**e** Distribution of risk score in patients stratified by IDH status and 1p/19q status. **f** The ROC curve to predict the Mesenchymal subtype according to risk score

The novel risk signature were further evaluated and validated in CGGA and TCGA datasets and the clinical information of patients were summarized in Table S1 (Additional file 1). We calculated the risk score of each patient by the formula and further detected its value in patients stratified by grade, subtype, MGMT promoter, IDH and 1p/19q status. As shown in Fig. 2, the risk score was up-regulated along with histological grades, and the increased expression was also observed in Mesenchymal, MGMT promoter unmethylated, LGG IDH-wildtype or GBM IDH-wildtype stratified patients. Similar observations were obtained in TCGA database (Additional file 2: Figure S1). Additionally, the receiving operator characteristic (ROC) curves for risk score and Mesenchymal subtype were performed and the area under the curve (AUC) were up to 89.6% (CGGA, Fig. 2) and 87.7% (TCGA, Additional file 2: Figure S1), respectively. This result suggested that the risk score may serve as a predictor for Mesenchymal subtype in glioma.

### Survival analysis and prognostic validity of the risk signature

**Fig. 3:**
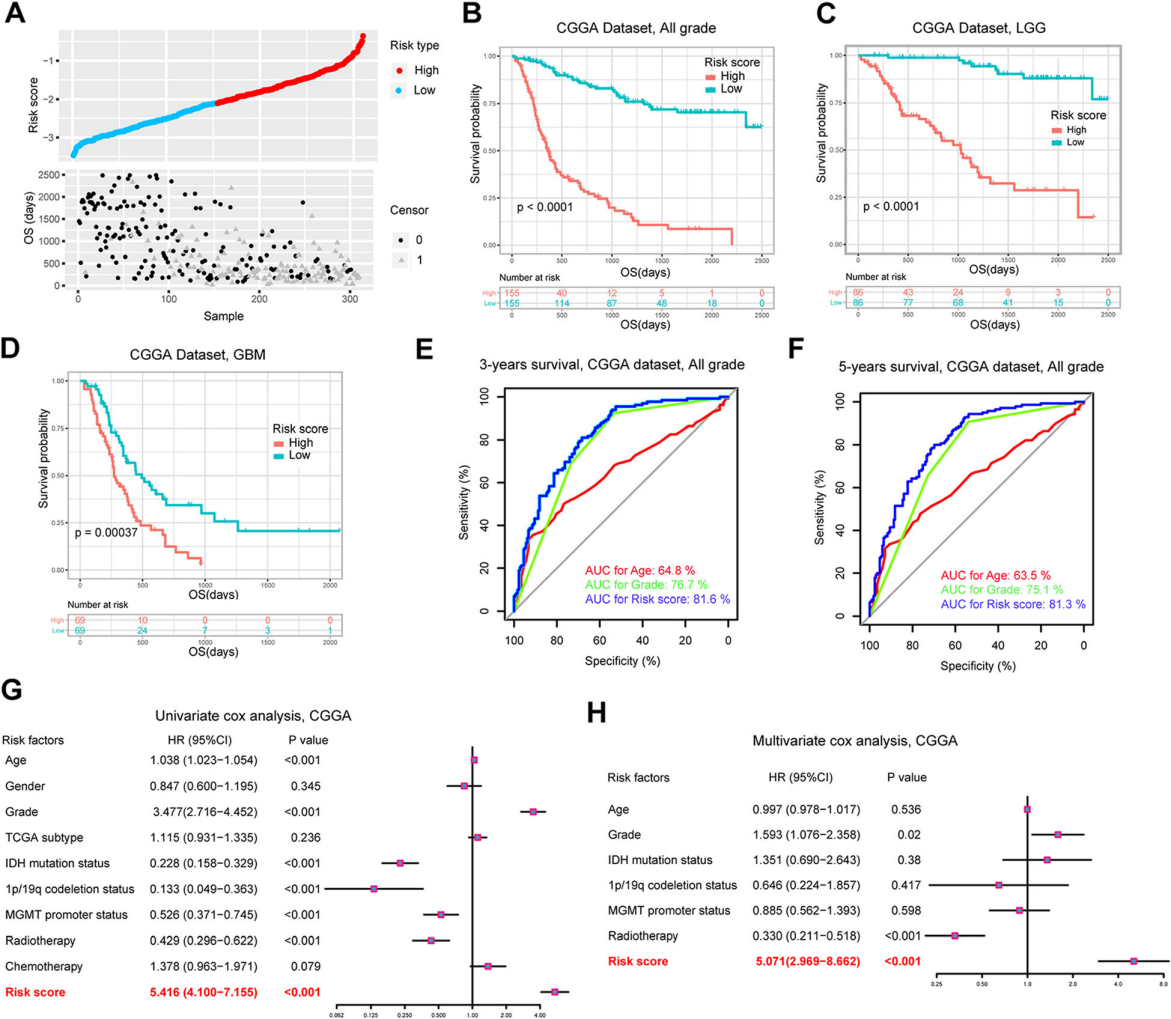
Survival analysis and prognostic validity of the risk signature in CGGA dataset. **a** The risk score distribution and survival overview of glioma patients. **b**-**d** Kaplan-Meier analyses of risk score for patient survival. **e** ROC analysis of age, grade and risk score for predicting 3-year survival of patients. **f** ROC analysis of age, grade and risk score for predicting 5-year survival of patients. **g**-**h** Univariate and multivariate Cox regression analyses of risk score and several other clinical pathologic features

To comprehensively investigate the risk signature, we evaluated the association of risk score and patient survival. Figure 3a showed the survival overview of each glioma patient in CGGA dataset and patients with high-risk score had poor prognosis. We further analyzed the high and low risk groups separately to clarify the relationship between risk score and molecular characteristics. Results revealed the enrichment of higher grades, wild type-IDH and Mesenchymal subtype in high risk groups, which indicated the consistency between high risk score and malignant molecular characteristics in glioma (Additional file 3: Table S2). Applying to the Kaplan-Meier survival analysis, we found that high risk score conferred reduced overall survival among diffuse glioma patients (*P* < 0.001, Fig. 3b-d). In particular, this risk signature could also be a good predictor of patient survival in GBM, an aggressive glioma subtype. Subsequently, we investigated the predictive accuracy of risk score in predication of 3- and 5-years OS by analyzing the ROC curves. Compared with traditional “age” and “grade” predictors, the risk signature showed favorable prognostic validity, with higher AUC up to 81.6 and 81.3% for 3- and 5-year survival, respectively, indicating its superior predictive value (Fig. 3e and f). The same results were validated in TCGA dataset (Additional file 3: Table S2 and Additional file 4: Figure S2).

To evaluate the prognostic value of risk signature across diffuse gliomas, we further performed univariate and multivariate Cox regression analysis. As shown in the results, risk score, age, WHO grade, IDH mutation status, MGMT promoter methylation status, 1p/19q deletion status and radiotherapy were significantly associated with patients’ survival in CGGA dataset (Fig. 3g). After further multivariable adjustment, the risk score remained an independent factor (*P* < 0.001), implying its powerful ability for predicting OS of glioma patients (Fig. 3h). Similar results were found in TCGA dataset (Additional file 4: Figure S2).

### Construction of an individualized prediction model

**Fig. 4:**
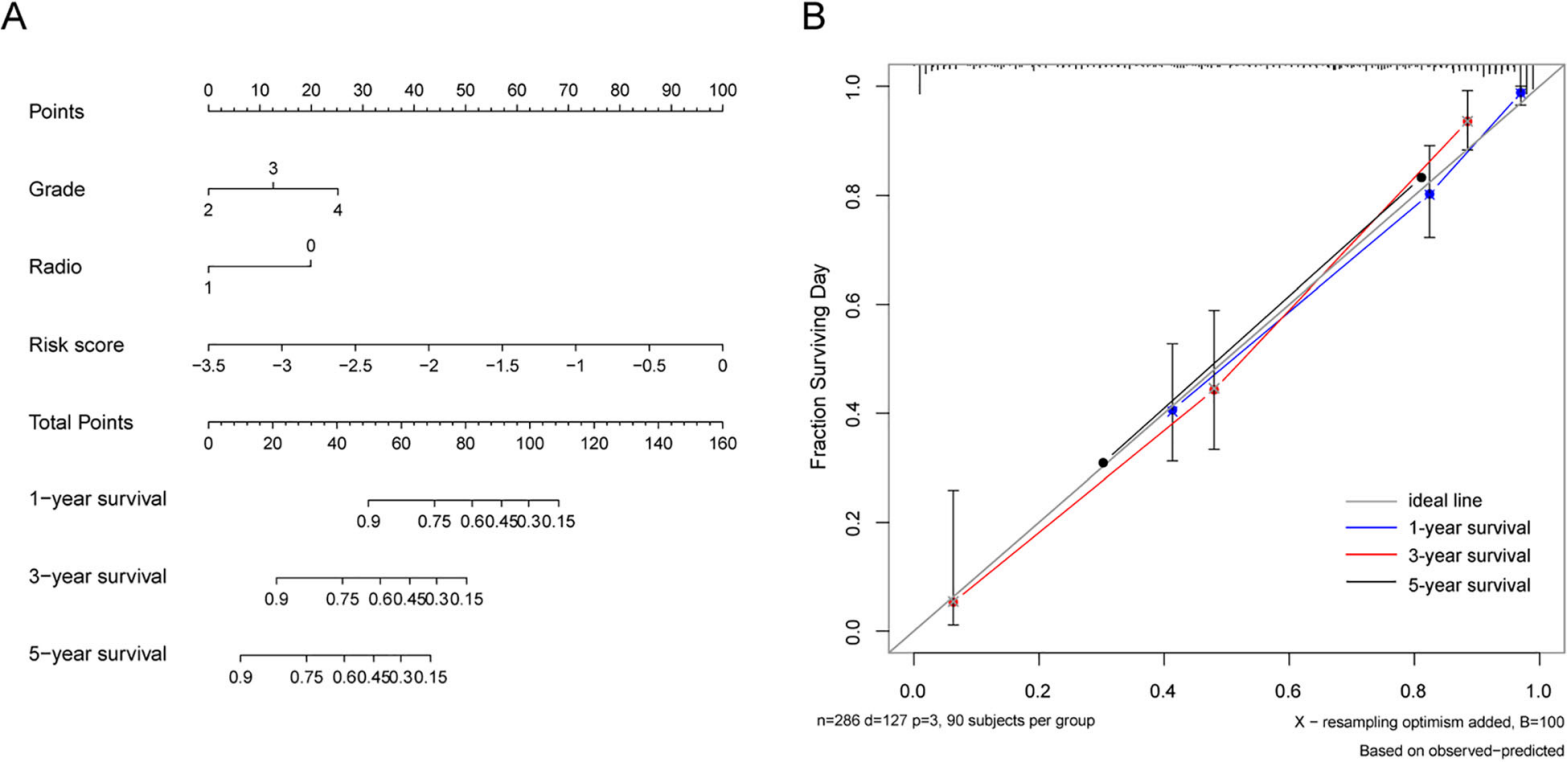
Nomogram model for predicting overall survival of patients in CGGA dataset. **a** A nomogram that integrates the signature risk score with the clinicopathologic characteristics. The ‘point’ represents the impact of each variable on patients’ survival. The line determines the ‘point’ received from the value of each variable. The sum of the individual points is presented as ‘total points’. The line drawn downward to the survival axis finally determines the likelihood of different survival rate. **b** The calibration curve for the nomogram model. Three colored lines (blue, red and black) represent the performance of the nomogram. A closer fit to the diagonal line (gray) indicates a better estimation

Using a backward stepwise method based on the smallest Akaike information criterion, the prognostic nomograms that integrated independent prognostic parameters were constructed. The results showed that signature risk score contributed the most risk points (range, 0–100), whereas the other clinical variables including grade and radiotherapy exhibited smaller contributions. The C-indices were 0.83 in the CGGA dataset and 0.85 in the TCGA cohort, indicating satisfactory concordance. The calibration curve also demonstrated excellent agreement between prediction and observation in the probabilities of 1-, 3-, and 5-year overall survival in both datasets (Fig. 4 and Additional file 5: Figure S3).

### Significant functions and pathway enrichment analysis

**Fig. 5:**
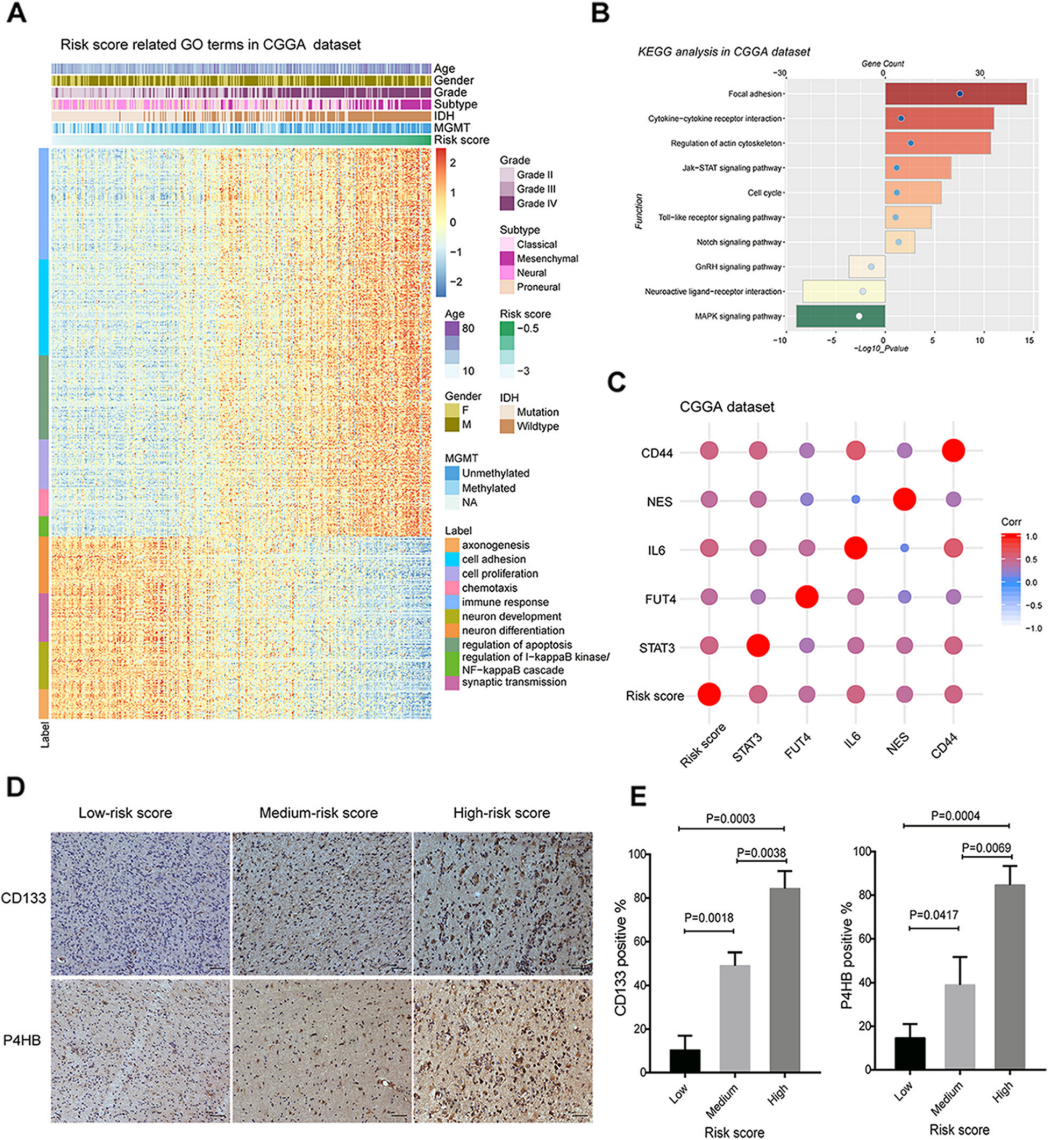
Biological function and pathway analysis in CGGA dataset. **a** Gene ontology analysis of the biological processes for risk score. **b** KEGG analysis of the enriched pathways for risk score. **c** Correlation between risk signature and CSC-related genes in glioma. **d** Representative images of IHC staining of CD133 and P4HB in glioma samples from the CGGA cohort. **d** Quantification of expression levels of CD133 and P4HB in IHC staining

To investigate the potential functions related to the gene signature, Pearson correlation analysis was performed to find out the genes that strongly correlated with risk score (Pearson |R| > 0.5) in CGGA and TCGA datasets. Afterwards the significant related genes were chosen for Gene Ontology analysis with online methods (DAVID, https://david.ncifcrf.gov/). As shown in the figures, the positive related genes were enriched in cell adhesion, proliferation, immune response, *etc* (Fig. 5a, Additional file 6: Figure S4). Both cell adhesion and proliferation can affect the drug resistance and stemness of cancer cells [16, 17]. The negatively related genes were more involved in normal neural development and physiological activity, such as neuron development, neuron differentiation, synaptic transmission, *etc* (Fig. 5a, Additional file 6: Figure S4). Additionally, we found that risk score positive related genes were mainly involved in the biological pathways, such as focal adhesion, regulation of actin cytoskeleton, cell cycle and Jak-STAT signaling pathway (Fig. 5b, Additional file 6: Figure S4).

To further validate the reliability, we investigate the relationships between risk signature and CSCs-related genes [18]. As shown in Fig. 5c, the significant association between risk score and CSCs-related genes were constructed using ‘ggcorrplot’ in R language. Results showed that the risk score was positively correlated with CSCs-related genes, suggesting that the higher the risk score, the higher the cancer cell stemness or/and drug resistance, which may indicate poor prognosis of glioma patients. The same results were obtained in the TCGA database (Additional file 6: Figure S4). To validate the results of bioinformatics analyses, we conducted IHC protein staining experiments to investigate the association of risk score and cancer cell stemness (*CD133*) and drug resistance (*P4HB*) in CGGA cohort. Representative IHC images were displayed in Fig. 5d. As expected, the expression levels of CD133 and P4HB increased in parallel with the risk score (Fig. 5e). Those results showed that high risk scores suggested high cell stemness and drug resistance of cancer cells.

### Application of the signature across diffuse gliomas

**Fig. 6:**
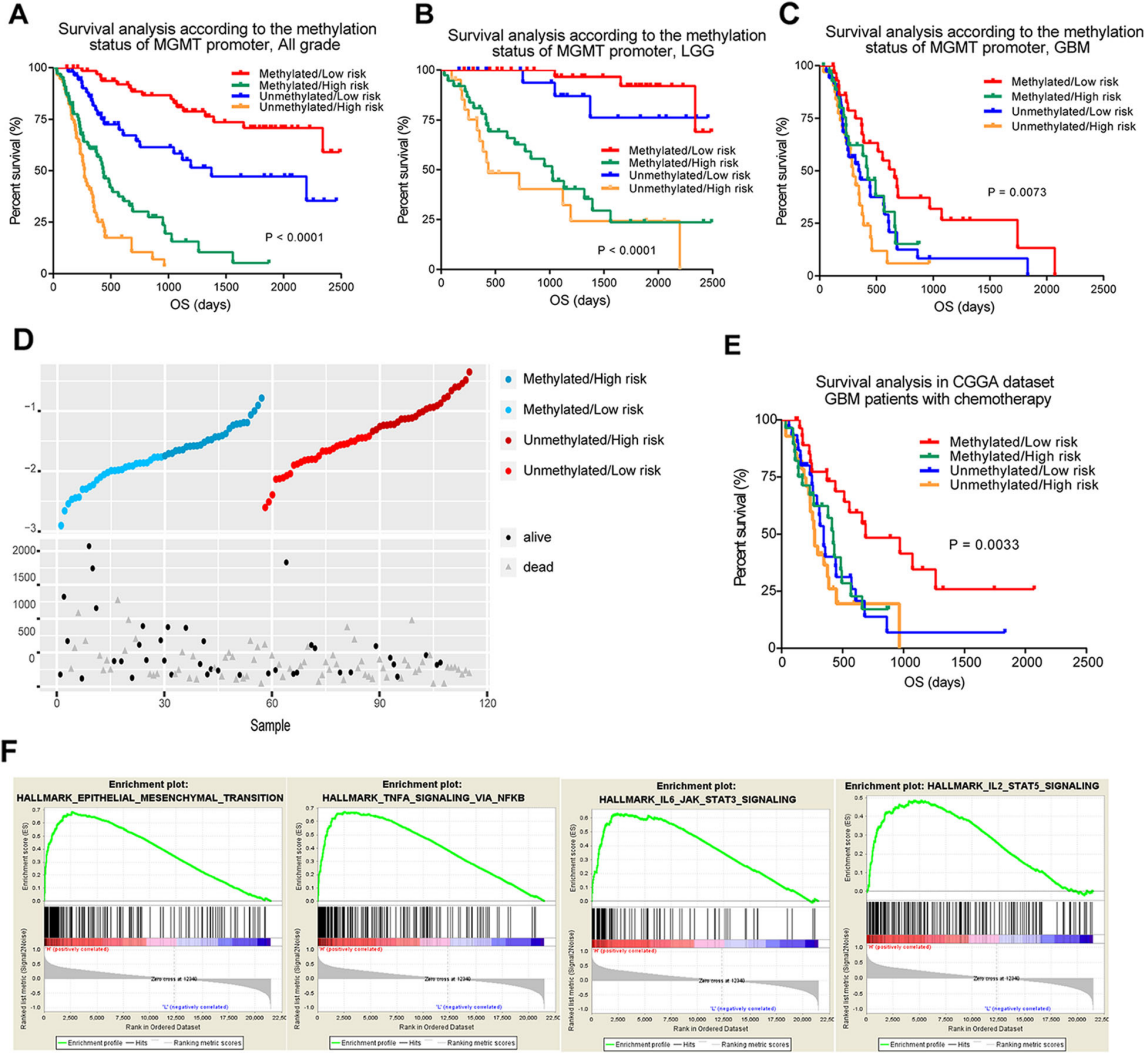
Application of the signature across diffuse gliomas in CGGA database. **a**-**c** Survival analysis of the four subgroups stratified according to risk signature and MGMT promoter methylation status across diffuse gliomas. **d**-**e** Survival distribution and Kaplan-Meier plots for OS of GBM patients with chemotherapy. **f** GSEA analysis based on the median value of risk score in GBM patients with chemotherapy

In the clinical treatment of glioma, the MGMT promoter methylation status is an important prognostic indicator, because patients with MGMT promoter methylation are prone to benefit from TMZ treatment. Therefore, we combined the methylation status of MGMT promoter to analyze the survival status of patients. As shown in Fig. 6a-c and Additional file 7: Figure S5, the survival analysis based on risk signature and MGMT promoter methylation status demonstrated remarkable stratification of the clinical courses into four subgroups. Patients with MGMT promoter unmethylation and high risk score had the worst prognosis, while patients with MGMT promoter methylation and low risk score had the best prognosis.

Especially, we subsequently focused on the GBM patients that treated with TMZ from CGGA dataset. According to the risk score and MGMT promoter methylation status, patients were divided into four subgroups. The risk score and overall survival distribution are shown in Fig. 6d. As shown, the risk score could well distinguish the prognosis of patients and a significant reduction in survival was observed in the high-risk group (Fig. 6e). We next conducted univariate and multivariate Cox analysis and revealed the independent correlation with OS and risk score, confirming its power for independently predicting prognosis (Table 1). The GSEA result showed the enrichment of epithelial mesenchymal transition, IL6-JAK-STAT3 pathway and TNFA signaling via NF-kB in the high-risk group (Fig. 6f), suggesting a potential mechanism of TMZ resistance. These findings were in accordance with the previous studies that the inhibition of NF-kB pathway or activated Stat3 could re-sensitize the resistant cells to therapeutic drugs [19, 20]. Collectively, we proved that this risk signature had a strong predictive effect on the prognosis of patients and could guide the individual treatment.

**Table 1:** Univariate and multivariate analysis of OS in CGGA RNA sequencing dataset, GBM patients wtith chemotherapy

Variables	Univariate analysis		Multivariate analysis	
	HR (95% CI)	*p* value	HR (95% CI)	p value
Risk score	2.533 (1.348–4.760)	0.004	2.578 (1.217–5.462)	0.013
Age at diagnosis	1.001 (0.974–1.028)	0.954	–	–
Gender	0.675 (0.353–1.293)	0.236	–	–
TCGA subtype	1.014 (0.789–1.304)	0.912	–	–
MGMT promoter status	0.539 (0.295–0.985)	0.045	0.759 (0.393–1.463)	0.410
IDH mutation status	0.657 (0.325–1.328)	0.242	–	–
Radiotherapy	0.303 (0.157–0.584)	< 0.001	0.759 (0.393–1.463)	< 0.001

### Potential mechanisms of drug resistance in glioma

**Fig. 7:**
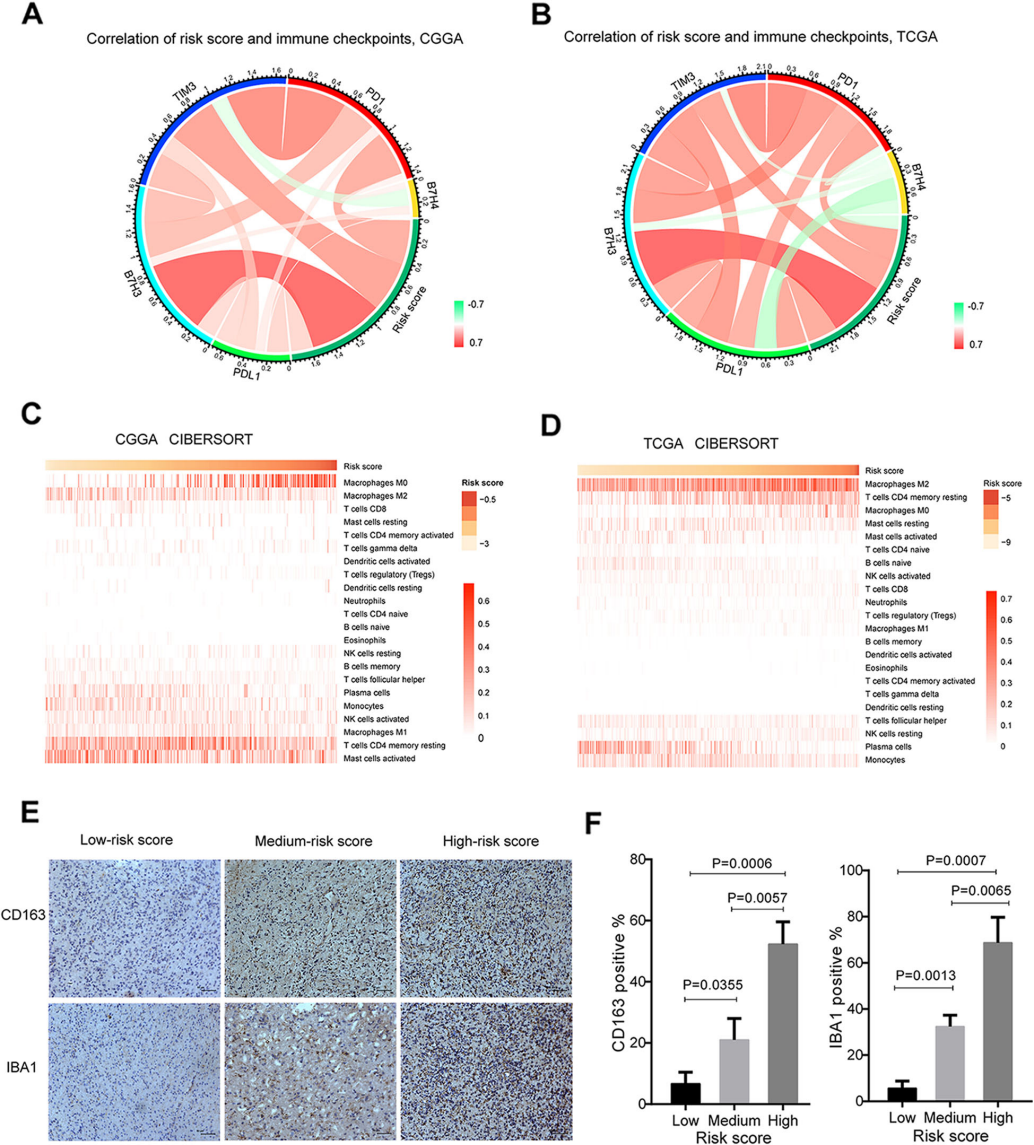
Relationship between drug resistance and immunity in patients with glioma. **a**-**b** Correlation between risk score and immune checkpoints in glioma from CGGA and TCGA datasets. **c**-**d** Component types of the immune cells infiltrated into glioma are analyzed with CIBERSORT. **e** Representative images of IHC staining of CD163 and IBA1 in glioma samples from the CGGA cohort. **f** Quantification of expression levels of CD163 and IBA1 in IHC staining

In the previous studies on the biological mechanism of this risk signature (Fig. 5a, Additional file 6: Figure S4) and patients after temozolomide treatment (Fig. 6f), it was suggested that drug resistance was related to immune response in glioma patients. It has also been reported that immune response and immune genes like B7H3 and Tim3 can contribute to the stemness and drug resistance [21, 22]. Then, the relationship between risk signature and immune checkpoints, including *B7H3*, *B7H4*, *Tim3*, *PD1*, and *PDL1* were analyzed [23, 24]. Our results demonstrated a tight connection between the risk score and *Tim3/B7H3* (Fig. 7a-b), which may partly explain the poor prognosis of patients and provide some clues for potential immunotherapy. We further estimated the abundance of various types of immune cell with CIBERSORT for CGGA and TCGA database (Fig. 7c-d). Samples with higher risk score exhibited apparent concordance with encirclement of macrophages in M0 phase, which have been reported to secrete immunosuppressive factors like tumor-supportive M2 macrophages in glioblastoma [25]. The results are consistent with the previously mentioned analysis that the enriched pathway containing immune factors after treatment with temozolomide (Fig. 6f). To further confirm these initial findings, we next studied the intratumoral immune cell infiltrates from glioma patients with IHC analysis and compared their immune cell profiles relative to the risk signature. As shown in the results, the expression levels of CD163 and IBA1 increased in parallel with the risk score (Fig. 7e-f). These results may explain the mechanism of drug resistance to some extent and provide new ideas for clinical treatment.

## Discussion

The origination and development of glioma is a complex biological process of multiple factors and steps. Due to the heterogeneity of glioma cells, the therapeutic efficacy of conventional treatment including surgery, chemotherapy and radiotherapy is limited [26]. After chemotherapy with temozolomide, the residual cancer stem cells and changes in gene expression of cancer cells may lead to drug resistance, thus leading to the failure of chemotherapy. In the present study, we developed a seven-gene-based signature to predict patients’ prognosis by integration analysis of TMZ-DR genes and CSC-related genes, and further reveal the potential mechanism of drug resistance. The risk signature is proved to be associated with poor prognosis of patients and its role as an independent prognosis predictor and individual survival estimation have been revealed.

The Gene Ontology analysis in our study have revealed that the positive genes associated with risk score were involved in cell proliferation, adhesion, chemotaxis and immune response, which have been demonstrated to induce the relapse of cancers [27, 28]. Moreover, the KEGG analysis has showed that Jak-stat signaling pathway and high risk score are closely related. The high level of stat activation is associated with Pan-cancers, especially for Stat3 and Stat5, which is mostly related to more dangerous tumors [29, 30]. The interaction network of risk signature and CSC-related genes indicated the relationship between cancer cell stemness and drug resistance. The hyperactivation of IL6/STAT3 pathway was reported to be essential for CSC self-renewal and tumorigenic [31, 32]. While other biomarkers, such as CD44 and Nestin, were correlated with tumor staging, tumor size and lymph node metastasis [33, 34]. The correlations between risk signature and cancer cell stemness and drug resistance have been confirmed by IHC assay. These findings may explain the poor prognosis of patients with high risk score to some extent. In addition, the further GSEA analysis in GBM patients with chemotherapy have found the enrichment of epithelial mesenchymal transition, IL6-JAK-STAT3 pathway and IL2-STAT5 pathway in high-risk group, indicating the important role of immune factors in tumor drug resistance. Further studies have shown the relationship between drug resistance and immune response, including immune checkpoints and immune cell infiltration in glioma [35–37]. Our findings have been validated in patient tissues and provide alternative therapeutic options for drug resistant patients in clinical application. These results are consistent with previous studies that drug resistance in glioma is associated with mesenchymal characteristics and immune responses [38–41].

The seven selected genes in signature have been studied and reported previously [42–44]. For instance, *TRIM67*, the most evolutionarily conserved member of tripartite motif (TRIM) family, was reported to function as a Ras inhibitor. The down regulation of *TRIM67* may contribute to the over-activation of Ras signal pathway and overgrowth of non-small cell lung cancer cells, leading to the malignant progression of tumor [45]. Moreover, *TRIM67* has been proved to play a necessary role in appropriate brain development and behavior [46]. In addition, Heyde et al. have investigated the resistance mechanism of monoclonal antibody drug trastuzumab in HER2-positive breast cancer. *NCAM2* and five other genes were significantly lower expressed in HCC1954 (drug resistance) than in BT474 (drug sensitivity) cell lines [47]. Other risk factors, such as *HPX* and *GRIA3*, have been reported in many cancers, including glioma, breast cancer and pancreatic cancer [48, 49]. These findings reported previously are consistent with our results in biological function analysis, implying the potential role of gene signature in brain functions and tumor progression.

The methylation status of MGMT promoter is an important factor in the clinical treatment of glioma patients. Combined with MGMT promoter methylation status, the signature can further divide patients into four subtypes. This classification is more conducive to clinical treatment. Additionally, this risk signature exhibited a good predictive value for the prognosis of GBM patients with chemotherapy. These results may provide some ideas for the follow-up treatment of patients. The current classification of gliomas is mainly based on grade, subtype, IDH mutation status and 1p/19q status. In this study, the signature could provide a perpendicular look on molecular characteristics to some extent, which means that high risk score implies malignant phenotypes, including higher grade, wild type-IDH and Mesenchymal subtype. Thus it might be reasonable to assume that we could reclassify gliomas based on the signature solely, where previous molecular characteristics have been taken into account in fact. However, our study is limited because it is retrospective, and should be further validated by prospective studies.

## Conclusions

In conclusion, we successfully built a novel gene signature using seven genes relevant to CSCs and TMZ resistance and reliably validated its molecular characteristics and clinical significance in two large sample-sized glioma datasets. The signature was proved to be an independent predictor in prognosis as the high risk scores always accompanied with shorter survival and more malignant phenotypes. Further functional analysis provided a clue for the underlying mechanisms in tumor progression and drug resistance. Our study revealed that the signature might be a potential prognostic biomarker and/or be used as therapeutic target in personalized clinical treatment in glioma patients.

## Supplementary information

**Additional file 1 : Table S1.** Clinical and molecular characteristics of patients in CGGA and TCGA datasets

**Additional file 2 : Figure S1.** Molecular characteristics and risk score distribution in patients from TCGA dataset. (A) Risk score distribution among different pathologic grades. (B) Risk score distribution among different subtypes of patients. (C) Risk score distribution between MGMT promoter methylated and unmethylated patients. (D-E) Distribution of risk score in patients stratified by IDH status and 1p/19q status. (F) The ROC curve to predict the Mesenchymal subtype according to risk score.

**Additional file 3 : Table S2**. Molecular characteristics of patients stratified by risk score in CGGA and TCGA datasets.

**Additional file 4 : Figure S2.** Survival analysis and prognostic validity of the risk signature in TCGA dataset. (A) The risk score distribution and survival overview of glioma patients. (B-D) Kaplan-Meier analyses of risk score for patient survival. (E) ROC analysis of age, grade and risk score for predicting 3-year survival of patients. (F) ROC analysis of age, grade and risk score for predicting 5-year survival of patients. (G-H) Univariate and multivariate Cox regression analyses of risk score and several other clinical pathologic features.

**Additional file 5 : Figure S3.** Nomogram model for predicting overall survival of patients in TCGA dataset. (A) A nomogram that integrates the signature risk score with the clinicopathologic characteristics. The ‘point’ represents the impact of each variable on patients’ survival. The line determines the ‘point’ received from the value of each variable. The sum of the individual points is presented as ‘total points’. The line drawn downward to the survival axis finally determines the likelihood of different survival rate. (B) The calibration curve for the nomogram model. Three colored lines (blue, red and black) represent the performance of the nomogram. A closer fit to the diagonal line (gray) indicates a better estimation.

**Additional file 6 : Figure S4.** Biological function and pathway analysis in TCGA dataset. (A) Gene ontology analysis of the biological processes for risk score. (B) KEGG analysis of the enriched pathways for risk score. (C) Correlation between risk signature and CSC-related genes in glioma.

**Additional file 7 : Figure S5.** Survival analysis of the four subgroups stratified according to risk signature and MGMT promoter methylation status in TCGA database.

## Data Availability

All the dataset and materials analyzed during this study were available.

## Abbreviations

AUC: The area under the curve
CGGA: Chinese Glioma Genome Atlas
CNS: Central nervous system
CSCs: Cancer stem cells
DAVID: Database for annotation,visualization and integrated discovery
GBM: Glioblastoma
GO: Gene ontology
GSEA: Gene set enrichment analysis
IC_50_: Half maximal inhibitory concentration
IDH: Isocitrate dehydrogenase
LASSO: Least absolute shrinkage and selection operator
LGG: Low-grade gliomas
OS: Overall survival
ROC: Receiver operating characteristic
TCGA: The Cancer Genome Atlas
TMZ: Temozolomide
TRIM: Tripartite motif
