# Supplementary material for: Comprehensive profiling identifies a novel signature with robust predictive value and reveals the potential drug resistance mechanism in glioma
Source: Cell Commun Signal. 2020 Jan 6;18:2. doi: 10.1186/s12964-019-0492-6 (PMC6943920; doi:10.1186/s12964-019-0492-6)
Supplement: Supplementary file 2 — Additional file 1 : Table S1. Clinical and molecular characteristics of patients in CGGA and TCGA datasets [file 12964_2019_492_MOESM1_ESM.docx]

Table S1. Clinical and molecular characteristics of patients in CGGA and TCGA datasets

| Characteristic | CGGA  (n=325) | TCGA  (n=699) |
| --- | --- | --- |
| Age |  |  |
| Mean (range) | 43.32 (8-81) | 47.34 (14-89) |
| Gender |  |  |
| Male | 203 (62%) | 368 (53%) |
| Female | 122 (38%) | 268 (38%) |
| NA | 0 | 63 (9%) |
| WHO Grade |  |  |
| II | 109 (34%) | 223 (32%) |
| III | 72 (22%) | 245 (35%) |
| IV | 144 (22%) | 168 (24%) |
| NA | 0 | 63 (9%) |
| TCGA subtype |  |  |
| Neural | 81 (25%) | 115 (16%) |
| Proneural | 102 (31%) | 250 (36%) |
| Classical | 74 (23%) | 92 (13%) |
| Mesenchymal | 68 (21%) | 105 (15%) |
| NA | 0 | 137 (20%) |
| IDH status |  |  |
| Mutation | 167 (51%) | 443 (63%) |
| Wild-type | 158 (49%) | 246 (35%) |
| NA | 0 | 10 (1.4%) |
| MGMT promoter status |  |  |
| Methylated | 139 (43%) | 492 (70%) |
| Unmethylated | 117 (36%) | 168 (24%) |
| NA | 69 (21%) | 39 (6%) |
| 1p/19q deletion |  |  |
| No | 218 (67%) | 520 (74%) |
| Yes | 36 (11%) | 172 (25%) |
| NA | 71 (22%) | 7 (1%) |
| Radiotherapy |  |  |
| Yes | 212 (65%) | NA |
| No | 84 (26%) |  |
| NA | 29 (9%) |  |
| Chemotherapy |  |  |
| Yes | 158 (49%) | NA |
| No | 128 (39%) |  |
| NA | 39 (12%) |  |
