# Supplementary material for: Comprehensive profiling identifies a novel signature with robust predictive value and reveals the potential drug resistance mechanism in glioma
Source: Cell Commun Signal. 2020 Jan 6;18:2. doi: 10.1186/s12964-019-0492-6 (PMC6943920; doi:10.1186/s12964-019-0492-6)
Supplement: Supplementary file 3 — Additional file 2 : Figure S1. Molecular characteristics and risk score distribution in patients from TCGA dataset. (A) Risk score distribution among different pathologic grades. (B) Risk score distribution among different subtypes of patients. (C) Risk score distribution between MGMT promoter methylated and unmethylated patients. (D-E) Distribution of risk score in patients stratified by IDH status and 1p/19q status. (F) The ROC curve to predict the Mesenchymal subtype according to risk score. [file 12964_2019_492_MOESM2_ESM.docx]

**Table S2.** Molecular characteristics of patients stratified by risk score in CGGA and TCGA datasets

| Characteristic | CGGA | | TCGA | |
| --- | --- | --- | --- | --- |
|  | Low-risk | High-risk | Low-risk | High-risk |
| WHO Grade |  |  |  |  |
| II | 94 (61%) | 11 (7%) | 180 (57%) | 42 (13%) |
| III | 38 (25%) | 29 (19%) | 135 (43%) | 108 (34%) |
| IV | 23 (14%) | 115 (74%) | 1 (0 %) | 167 (53%) |
| NA | 0 | 0 | 0 | 0 |
| TCGA subtype |  |  |  |  |
| Neural | 67 (43%) | 9 (6%) | 87 (28%) | 17 (5%) |
| Proneural | 69 (45%) | 30 (20%) | 160 (50%) | 67 (21%) |
| Classical | 12 (8%) | 58 (37%) | 1 (0%) | 88 (28%) |
| Mesenchymal | 7 (4%) | 58 (37%) | 5 (2%) | 95 (30%) |
| NA | 0 | 0 | 63 (20%) | 50 (16%) |
| IDH status |  |  |  |  |
| Mutation | 124 (80%) | 40 (26%) | 295 (93%) | 93 (30%) |
| Wild-type | 31 (20%) | 115 (74%) | 19 (6%) | 217 (68%) |
| NA | 0 | 0 | 2 (1%) | 7 (2%) |
| MGMT promoter status |  |  |  |  |
| Methylated | 73 (47%) | 63 (41%) | 282 (89%) | 157 (50%) |
| Unmethylated | 36 (23%) | 75 (48%) | 34 (11%) | 121 (38%) |
| NA | 46 (30%) | 17 (11%) | 0 | 39 (12%) |
| 1p/19q deletion |  |  |  |  |
| No | 98 (63%) | 120 (77%) | 165(52%) | 308 (97%) |
| Yes | 31 (20%) | 5 (3%) | 151 (48%) | 2 (1%) |
| NA | 26 (17%) | 30 (20%) | 0 | 7 (2%) |
