# Supplementary figures and images for: Comprehensive profiling identifies a novel signature with robust predictive value and reveals the potential drug resistance mechanism in glioma
Source: Cell Commun Signal. 2020 Jan 6;18:2. doi: 10.1186/s12964-019-0492-6 (PMC6943920; doi:10.1186/s12964-019-0492-6)

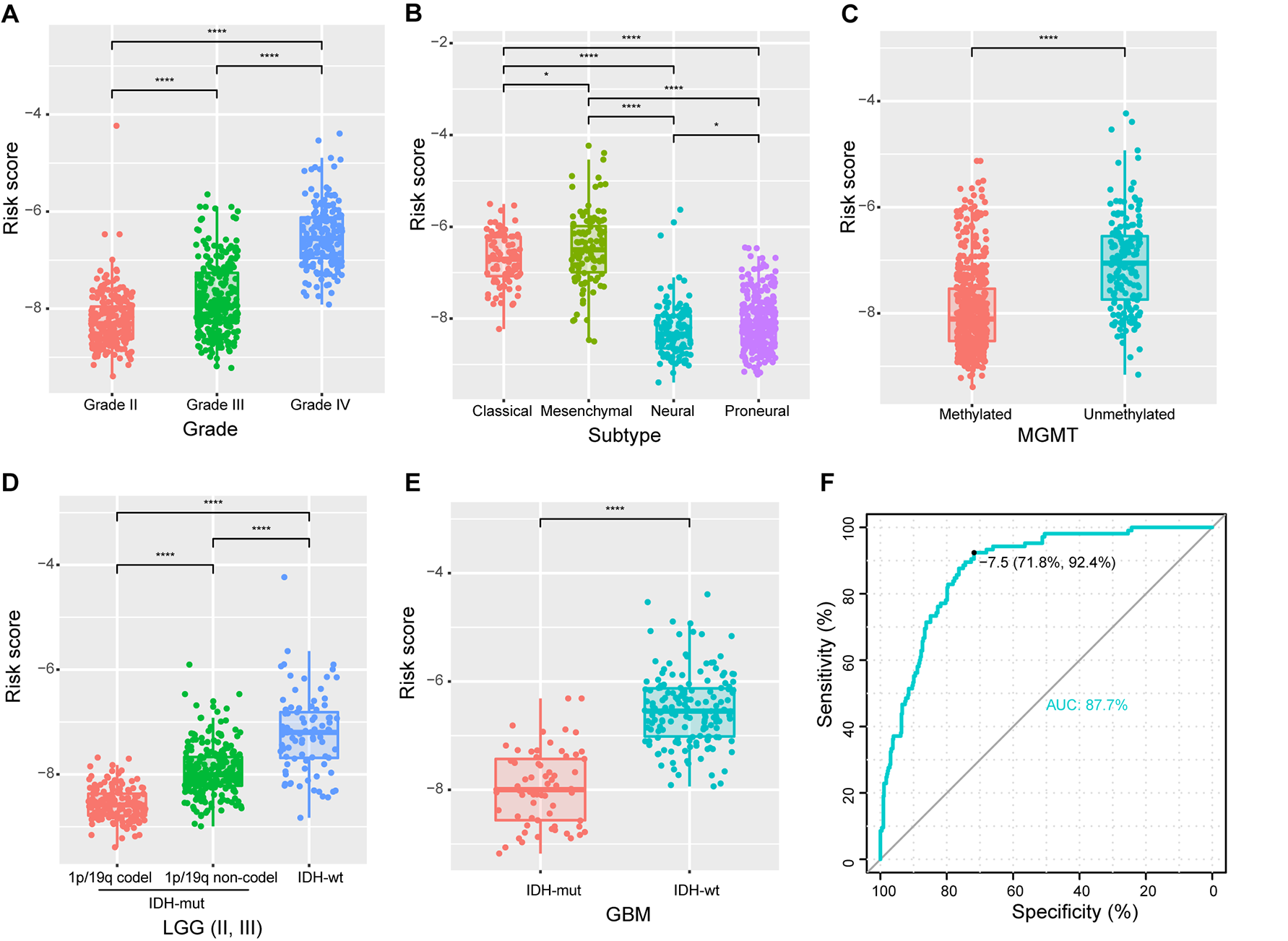

Supplement: Supplementary file 4 — Additional file 3 : Table S2. Molecular characteristics of patients stratified by risk score in CGGA and TCGA datasets. [file 12964_2019_492_MOESM3_ESM.tif]

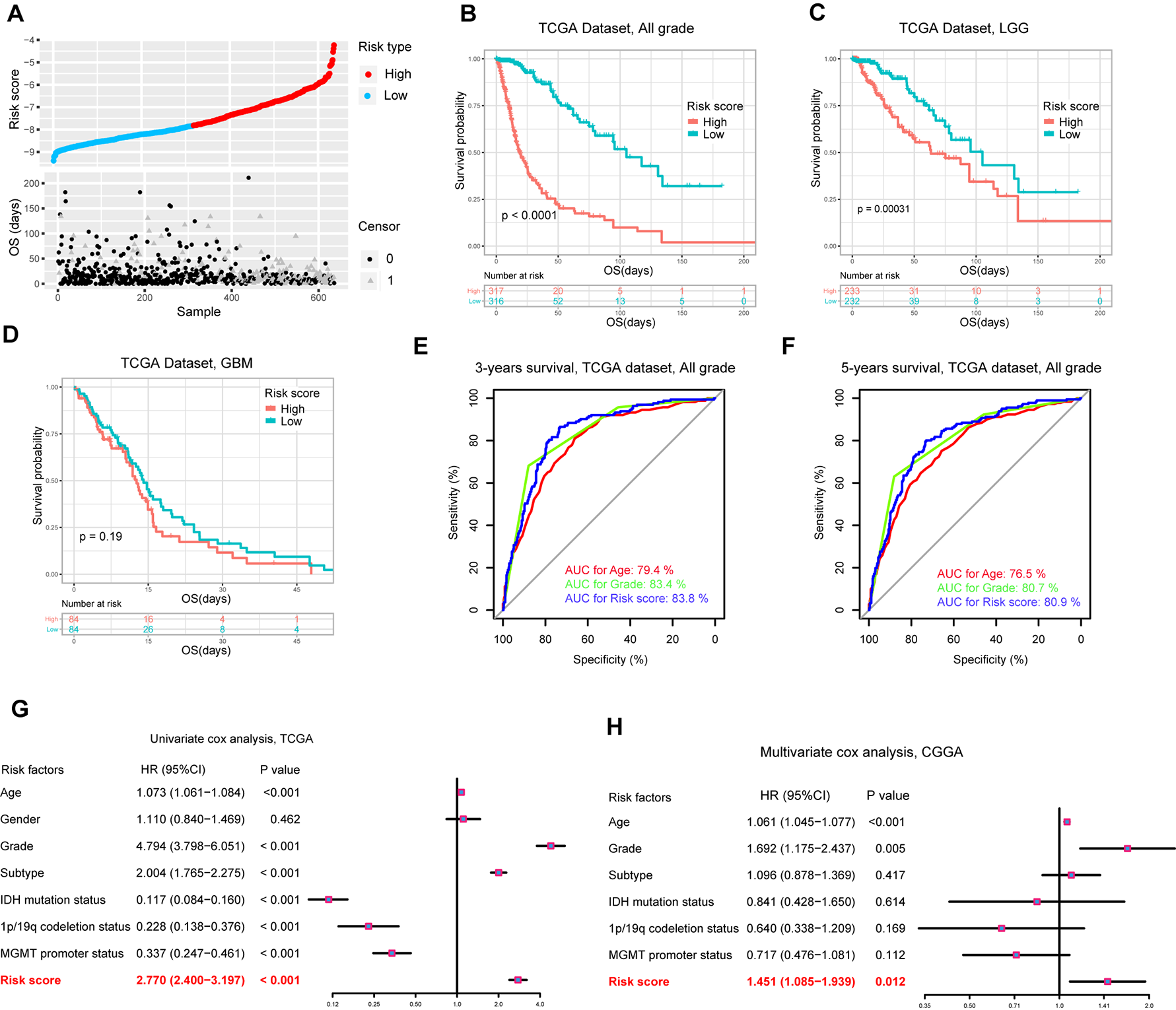

Supplement: Supplementary file 5 — Additional file 4 : Figure S2. Survival analysis and prognostic validity of the risk signature in TCGA dataset. (A) The risk score distribution and survival overview of glioma patients. (B-D) Kaplan-Meier analyses of risk score for patient survival. (E) ROC analysis of age, grade and risk score for predicting 3-year survival of patients. (F) ROC analysis of age, grade and risk score for predicting 5-year survival of patients. (G-H) Univariate and multivariate Cox regression analyses of risk score and several other clinical pathologic features. [file 12964_2019_492_MOESM4_ESM.tif]

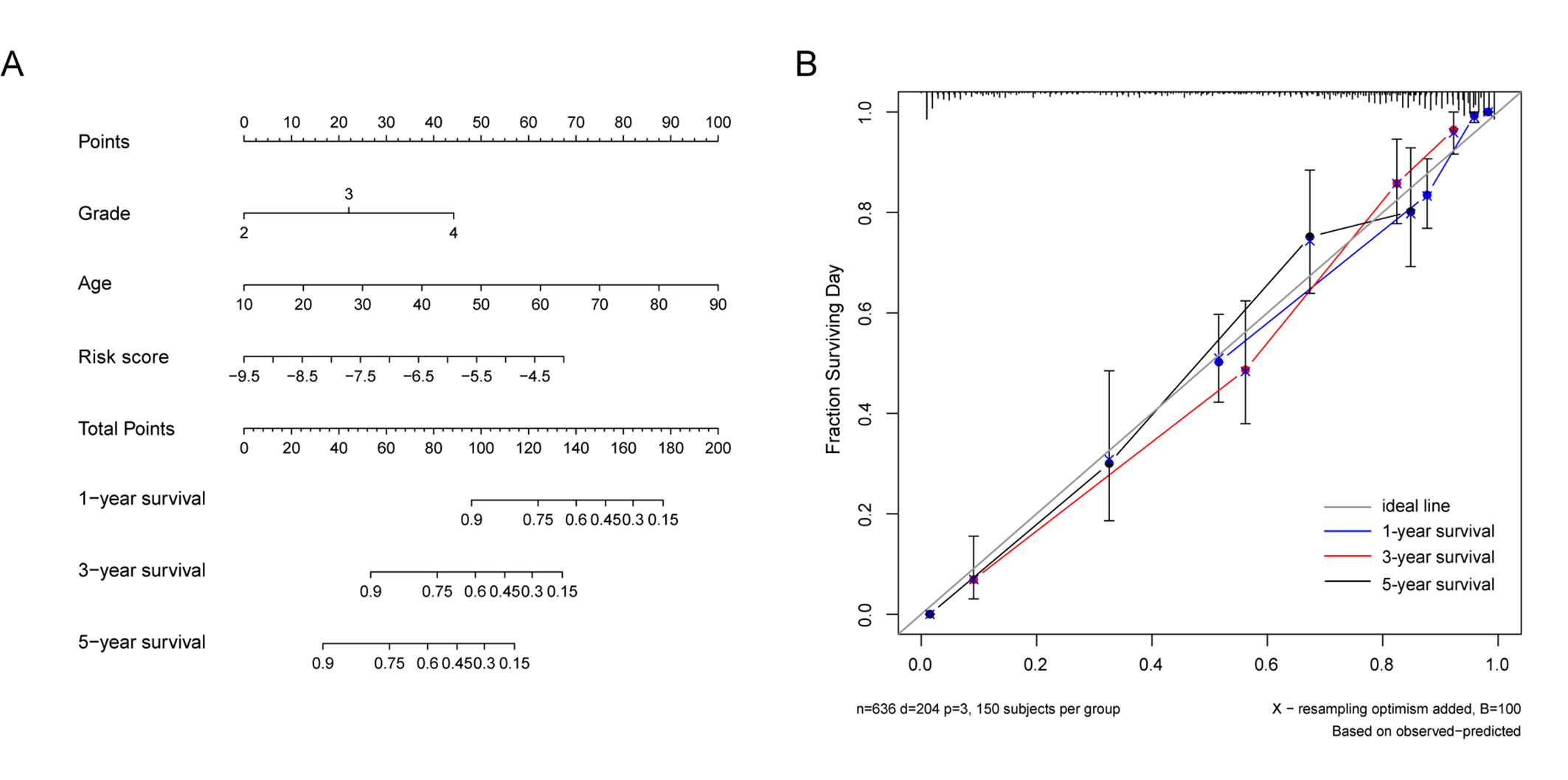

Supplement: Supplementary file 6 — Additional file 5 : Figure S3. Nomogram model for predicting overall survival of patients in TCGA dataset. (A) A nomogram that integrates the signature risk score with the clinicopathologic characteristics. The ‘point’ represents the impact of each variable on patients’ survival. The line determines the ‘point’ received from the value of each variable. The sum of the individual points is presented as ‘total points’. The line drawn downward to the survival axis finally determines the likelihood of different survival rate. (B) The calibration curve for the nomogram model. Three colored lines (blue, red and black) represent the performance of the nomogram. A closer fit to the diagonal line (gray) indicates a better estimation. [file 12964_2019_492_MOESM5_ESM.tif]

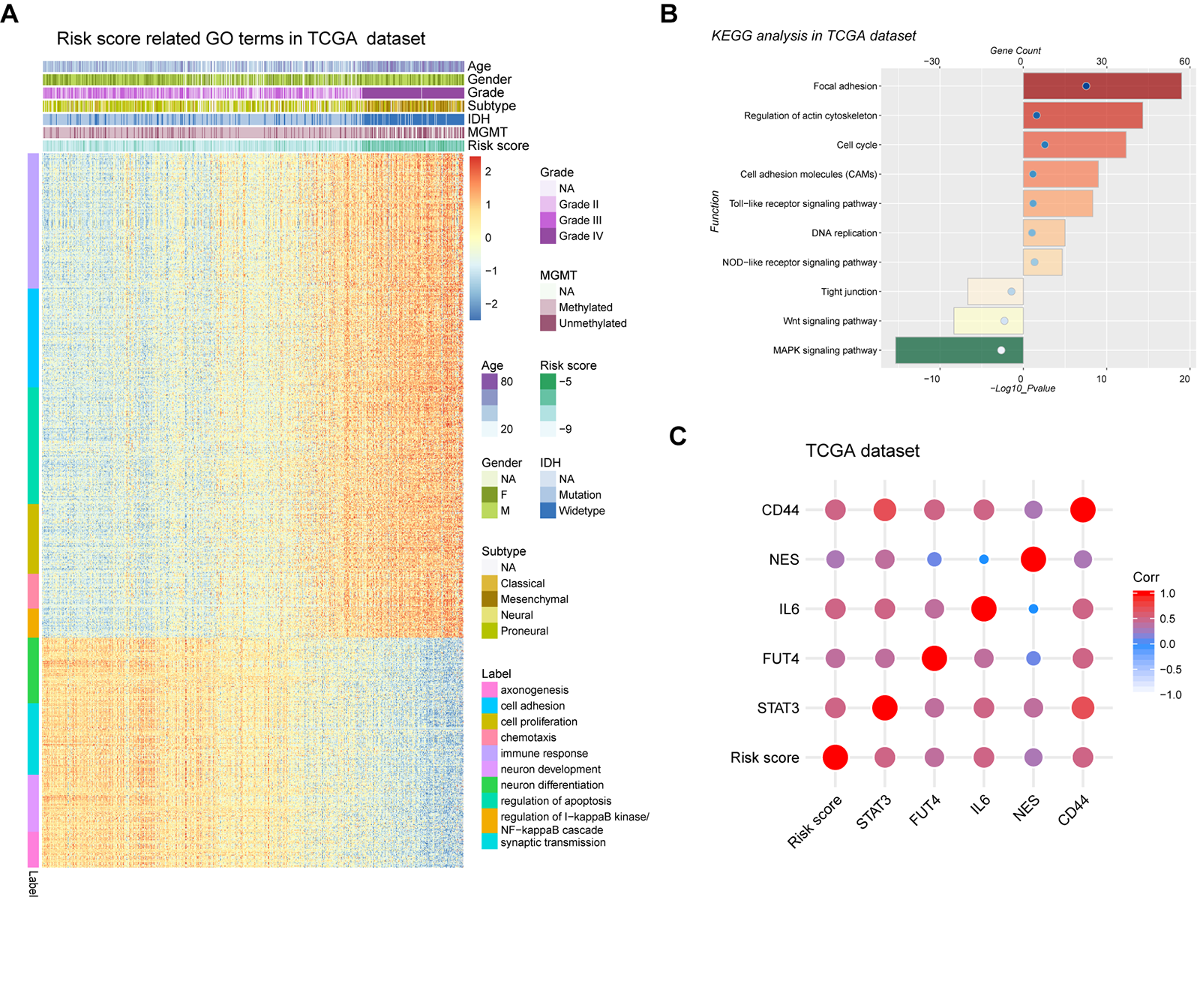

Supplement: Supplementary file 7 — Additional file 6 : Figure S4. Biological function and pathway analysis in TCGA dataset. (A) Gene ontology analysis of the biological processes for risk score. (B) KEGG analysis of the enriched pathways for risk score. (C) Correlation between risk signature and CSC-related genes in glioma. [file 12964_2019_492_MOESM6_ESM.tif]

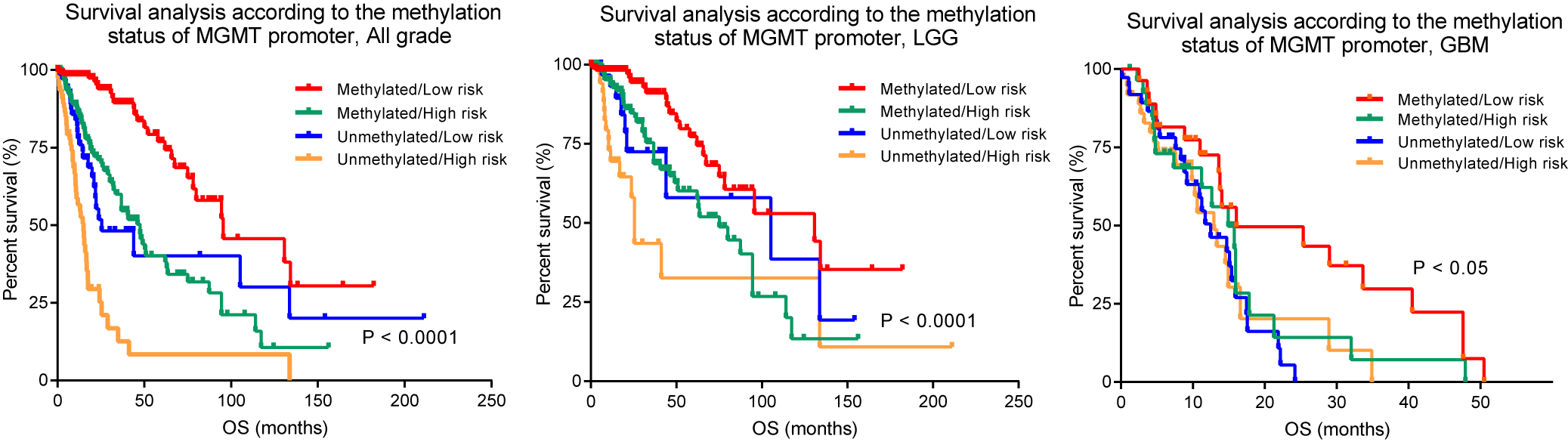

Supplement: Supplementary file 8 — Additional file 7 : Figure S5. Survival analysis of the four subgroups stratified according to risk signature and MGMT promoter methylation status in TCGA database. [file 12964_2019_492_MOESM7_ESM.tif]
